# Viability Reagent, PrestoBlue, in Comparison with Other Available Reagents, Utilized in Cytotoxicity and Antimicrobial Assays

**DOI:** 10.1155/2013/420601

**Published:** 2013-04-04

**Authors:** Namrita Lall, Cynthia Joan Henley-Smith, Marco Nuno De Canha, Carel Basson Oosthuizen, Danielle Berrington

**Affiliations:** Department of Plant Science, University of Pretoria, Pretoria 0002, South Africa

## Abstract

This study compared different commercially available viability reagents. The growth indicator reagents include *p*-iodonitrotetrazolium violet (INT), PrestoBlue, and Alamar Blue which were used for antimicrobial analysis against *Streptococcus mutans*, *Prevotella intermedia*, *Propionibacterium acnes*, and *Mycobacterium tuberculosis*. PrestoBlue and Alamar Blue are resazurin based reagents that resulted in a quick and easily distinguishable colour change that allowed for visual readings. INT and Sodium 3′-[1-(phenyl amino-carbonyl)-3,4-tetrazolium]-bis-[4-methoxy-6-nitro] benzene sulfonic acid hydrate (XTT) are tetrazolium based reagents which are converted to a formazan dye in the presence of metabolically active mitochondria enzyme. For cell viability analysis, reagents XTT and PrestoBlue were compared. PrestoBlue was able to clearly indicate the minimum inhibitory concentration (MIC) of various positive drug controls on various microbial strains. PrestoBlue was also a good indicator of the 50% inhibitory concentration (IC_50_) of positive drug controls on various cell lines.

## 1. Introduction

PrestoBlue and Alamar Blue reagents are resazurin based, membrane permeable solutions that upon reduction form resorufin, a red fluorescent compound which can be quantitatively measured to determine viability. Initially developed as a cell viability indicator, PrestoBlue has been indicated for use on nonmammalian cells, such as bacteria, yeast, and eukaryotic cells. The variable reading methods of PrestoBlue makes this reagent an attractive alternative in cellular and microbiology. PrestoBlue can be measured either visually, using absorbance or utilising the fluorescent outputs of the reduced resorufin [1].

*p*-iodonitrotetrazolium violet (INT) is a tetrazolium dye precursor that once reduced forms a purple formazan dye (Sigma-Aldrich). Sodium 3′-[1-(phenyl amino-carbonyl)-3,4-tetrazolium]-bis-[4-methoxy-6-nitro] benzene sulfonic acid hydrate (XTT) is also a tetrazolium based reagents which in the presence of metabolically active cells reduces the tetrazolium salt to an orange coloured formazan compound. The reduction of the tetrazolium salt is due to the activity of the mitochondria enzyme in the active cells. The intensity of the formazan compound can be measured using absorbance where the intensity of the compound is directly proportional to the number of metabolically active cells [2].

The aim of this study was to validate the use of PrestoBlue as a growth indicator and cell viability reagent by comparing it to other similar commercially available reagents.

## 2. Materials and Methods

### 2.1. Chemicals and Reagents

All microbial strains were purchased from American Type Culture Collection (ATCC), MD, USA. The A431 cell line and HeLa cell line were purchased from ECACC and Highveld Biological (Pty) Ltd., respectively. INT was obtained from Sigma-Aldrich, South Africa. PrestoBlue and Alamar Blue were both purchased from Invitrogen Corporation, San Diego, USA. XTT cell proliferation Kit II was obtained from Roche Applied Sciences, South Africa. The cell culture medium, trypsin-EDTA, fetal bovine serum (FBS), phosphate buffer saline (PBS), and antibiotics were supplied by Highveld Biological (Pty) Ltd. (Modderfontein, Johannesburg, RSA). All other reagents were of analytical grade.

### 2.2. Determination of the Minimum Inhibitory Concentration (MIC) for the Microbial Strains

#### 2.2.1. Culturing of Microorganisms

The microorganisms used in this study included *Prevotella intermedia* (ATCC 25611), *Streptococcus mutans* (ATCC 25175), and *Propionibacterium acnes *(ATCC 11827). *S. mutans *and *P. intermedia* were grown on Casein-peptone Soymeal-peptone Agar medium (CASO) enriched with 1% sucrose (Merck Chemicals (Pty) Ltd., Wadeville, South Africa) under anaerobic conditions in an anaerobic jar with Anaerocult A (Merck KGaA, Darmstadt, Germany) at 37°C for 48 hrs. Subculturing was done every second week. Inoculants were prepared by suspending bacterial test organisms in quarter strength sterile Ringer solution (Merck KGaA, Darmstadt, Germany) until turbidity was compatible with McFarland Standard 1 (Merck Chemicals (Pty) Ltd., Wadeville, South Africa). Furthermore a bacterial concentration of 4 × 10^8^ (colony forming units) CFU/mL for *P. intermedia* and 3 × 10^8^ CFU/mL for *S. mutans *was used [3].

*P. acnes *was cultured on Tryptone Soy Agar (Merck Chemicals (Pty) Ltd., Wadeville, South Africa) under anaerobic conditions in an anaerobic jar with Anaerocult A at 37°C for 72 hrs. Subculturing was done every 4th week as the doubling time of the bacterium is slow. Inoculants were prepared by suspending the bacterial colonies in Nutrient Broth (Merck Chemicals (Pty) Ltd., Wadeville, South Africa) until turbidity was compatible with a McFarland Standard 0.5 at a bacterial concentration of 1.5 × 10^8^ CFU/mL [4].

#### 2.2.2. Evaluation of MIC Values for Microorganisms

The microdilution technique using 96-well microtitre plates, as described by Eloff [5], was used to obtain the MIC values of the positive drug controls against the various microorganisms. The positive controls for oral bacteria, chlorhexidine gluconate (CHX) (Dental Warehouse, Sandton, South Africa) at 12.5 *µ*g/mL, and Tetracycline (Sigma-Aldrich, 3050 Spruce Street, St. Louis) at 200 *μ*g/mL for *P. acnes* were serially diluted in relevant broth medium adding 48 h old oral bacteria and 72 h old *P. acnes* and were incubated at 37°C in anaerobic conditions. The final concentration of CHX, ranged 3.13 *µ*g/mL–2.44 × 10^−2^ *µ*g/mL and Tetracycline ranged 100 *μ*g/mL–0.781 *μ*g/mL. *S. mutans* and *P. intermedia* were incubated for 24 hrs and *P. acnes *for 72 hrs at 37°C.

To indicate bacterial growth, 40 *μ*L of (0.2 mg/mL) INT, 20 *μ*L PrestoBlue, and 20 *μ*L Alamar Blue was added to microplate wells and reincubated until a colour change occurred. The MIC was defined as the lowest concentration that inhibited the growth of the bacteria [6].

#### 2.2.3. Evaluation of MIC against *Mycobacterium tuberculosis*

For the antimycobacterial assay the H37Rv (ATCC 27264) *M. tuberculosis* strain was used. The bacteria were cultured on Löwenstein-Jensen (LJ) medium (SA Medical Research Council, Pretoria) for three weeks (37°C, 5% CO_2_). One colony was transferred under sterile conditions to 50 mL of 7H9 broth media (Sigma-Aldrich, South Africa), 10% OADC (Sigma-Aldrich, South Africa) and 2% PANTA (Becton, Dickinson and Company, USA). The bacteria were further cultured for one week. After the final incubation period, the bacteria were adjusted in 7H9 broth media to a turbidity of a McFarland Standard 1. Bacterial suspensions were further diluted 1 : 25 to obtain a concentration of 2 × 10^8^ CFU/mL [7].

The Microtitre Alamar Blue assay (MABA) and Microtitre PrestoBlue assay (MPBA) were used to determine the MIC of positive drug controls on the bacteria [7]. Standard antituberculosis agents, INH (Sigma-Aldrich, Becton, Dickinson and Company), and RIF (Sigma-Aldrich, Becton, Dickinson and Company) were used. Stock solutions were diluted in 7H9 broth media to a final assay concentrations which ranged 5 *µ*g/mL–0.078 *µ*g/mL and 6 *µ*g/mL–0.1875 *µ*g/mL for INH and RIF, respectively. A 2% dimethyl sulfoxide (DMSO) solvent control, media control, and bacterial control was included in the assay. One hundred microlitres of bacteria was added to the inner wells of the 96-well plates. The outer perimeter wells were used to compensate for evaporation by adding 200 *µ*L of dH_2_O. The plates were incubated for 5 to 7 days at 37°C, 5% CO_2_. After 5 days 20 *µ*L of Alamar Blue/PrestoBlue indicator solution was added to one of the bacterial control wells. If a colour change was observed, all the subsequent wells received 20 *µ*L of indicator solution. If no colour change was observed the plates were incubated for further 24 hrs.

### 2.3. Comparison of Growth Indicators for Cytotoxicity Analysis

The HeLa and A431 cell lines were maintained in Eagle's Minimum Essential Medium supplemented with 10% FBS and 1% antibiotics (100 U/mL penicillin, 100 *µ*g/mL streptomycin) and 250 *µ*g/mL fungizone. The cells were grown at 37°C in a humidified incubator set at 5% CO_2_. Cells were subcultured by treating them with trypsin-EDTA (0.25% trypsin containing 0.01% EDTA) for 10 minutes.

Cytotoxicity was measured using the XTT cell proliferation Kit II and MPBA. The method described by Zheng et al. [8] was used to perform the assay. Both cell lines were seeded in a 96-well microtitre plate at a concentration of 1 × 10^5^ cells/mL. Cells were allowed to attach for 24 hrs at 37°C and 5% CO_2_. The cells were exposed to the positive drug control Actinomycin D (Sigma-Aldrich, South Africa) with concentrations ranging between 0.5 *µ*g/mL and 0.002 *µ*g/mL. The microtitre plate was incubated for further 72 hrs and thereafter 50 *µ*L XTT was added to a final concentration of 0.3 mg/mL to one set of plates and 20 *µ*L PrestoBlue was added to another set of plates. The plates were incubated for further 2 hrs where after the absorbance of the colour complex was read at 490 nm with a reference wavelength set at 690 nm for XTT and at 570 nm with a reference wavelength set at 600 nm for PrestoBlue, using a BIO-TEK Power-Wave XS multiwell plate reader.

## 3. Results and Discussion

### 3.1. Determination of MIC for Microbial Strains

#### 3.1.1. Evaluation of MIC against Oral Microorganisms

**Figure 1:**
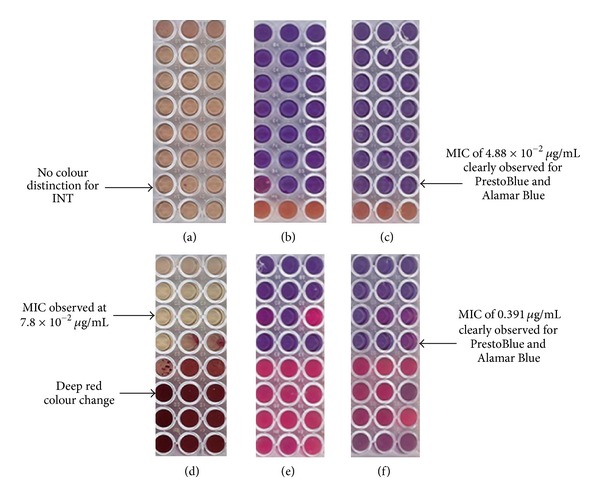
Comparison of different growth indicators on oral microorganisms, (a) INT on *S. mutans,* (b) PrestoBlue on *S. mutans,* (c) Alamar Blue on *S. mutans,* (d) INT on *P. intermedia,* (e) PrestoBlue on *P. intermedia* and (f) Alamar Blue on *P. intermedia. *

The colour change of the growth indicators INT, PrestoBlue, and Alamar Blue for *S. mutans* and *P. intermedia* is depicted in Figure 1.

The development time for each of the oral bacteria differed. With the addition of INT, *P. intermedia* developed within 40 minutes; however, *S. mutans* development took over two hours and it was difficult to distinguish where the MIC occurred. Conversely with PrestoBlue or Alamar Blue, *S. mutans* developed immediately and the MIC was easily distinguishable, with pink indicating viable bacteria and blue indicating nonviable bacteria. *P. intermedia* developed within 20 minutes, although the colour change took slightly longer with Alamar Blue. The MIC values obtained for the inhibition of *S. mutans* and *P. intermedia* by CHX showed the same values using both PrestoBlue and Alamar Blue at 4.88 × 10^−2^ *µ*g/mL and 0.391 *µ*g/mL, respectively. *P. intermedia* showed a slightly higher MIC with the development of INT (at 0.78 *µ*g/mL). It was almost impossible to determine the MIC value for the inhibition of *S. mutans* using INT. There appeared to be a distinctive colour change for each of the bacteria whether using PrestoBlue or Alamar Blue.

#### 3.1.2. Evaluation of MIC against *Propionibacterium acnes*

**Figure 2:**
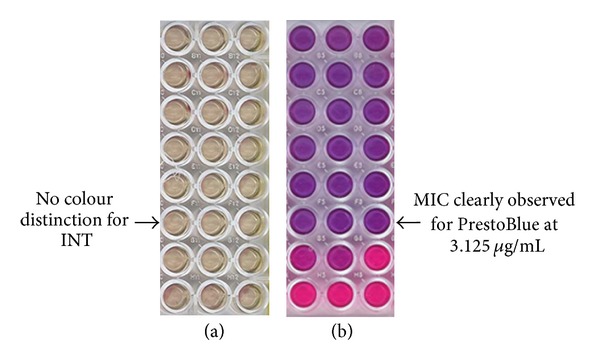
Comparison of different growth indicators on *P. acnes*: (a) INT and (b) PrestoBlue on *P. acnes*.

The colour change of the growth indicators INT and PrestoBlue for *P. acnes* were observed after addition of the reagents as seen in Figure 2.

The comparison between INT and PrestoBlue for determination of MIC values for bacterial cells treated with tetracycline was clearly indicated with the use of PrestoBlue when compared with INT. The *P. acnes *bacteria had a tendency to coagulate at the bottom of the wells during the incubation period and this was where the INT was reduced to the violet colour. The PrestoBlue provided an advantage in that the entire well was coloured, making visualisation of MIC values easier. Tetracycline treated cells in the presence of INT showed no colour change as seen in Figure 2(a). However in the presence of PrestoBlue, there was a clear distinction between wells with metabolically active bacterial cells (pink) and those which had been inhibited (blue) by the tetracycline control drug. Although the incubation period of the PrestoBlue plates was slightly longer (±3 hrs) compared with INT (±1 h) the determination of MIC values was much clearer. The MIC of tetracycline was determined to be 3.125 *µ*g/mL with PrestoBlue and could not be visually determined with INT.

#### 3.1.3. Evaluation of MIC against *Mycobacterium tuberculosis*

The health risk involved in working with *M. tuberculosis* meant no clear pictures of the colour changes could be obtained.

Due to the slow growth rate of *M. tuberculosis*, the conversion of the Alamar Blue and PrestoBlue into a red colour took up to 24 hrs. Although a colour change could be observed after 3 hrs, the longer incubation period resulted in a more distinguishable separation between viable pink bacterial wells and blue inhibited wells. There were no differences observed between the MIC values of the positive controls when comparing Alamar Blue and PrestoBlue. INH showed an MIC of 0.13 *µ*g/mL whereas Rifampicin showed an MIC of 0.75 *µ*g/mL for both PrestoBlue and Alamar Blue. The PrestoBlue had a slightly faster conversion rate compared to the Alamar Blue, but at 24 hr incubation the readings of the MICs were the same.

### 3.2. Comparison of Growth Indicators for Cytotoxicity Analysis

**Figure 3:**
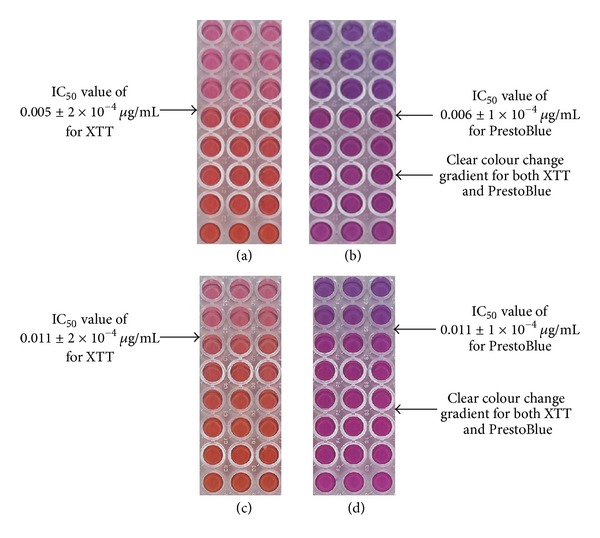
Comparison of different growth indicators on various cell lines (a) XTT on HeLa cells, (b) PrestoBlue on HeLa cells, (c) XTT on A431 cells, and (d) PrestoBlue on A431 cells.

XTT and PrestoBlue were used to determine the antiproliferative effect of Actinomycin D on HeLa cells and A431 cells as seen in Figure 3.

For both growth indicators a colour change was observed after approximately 2 hrs. Both cell lines were exposed to Actinomycin D and the IC_50_ values were determined.

The IC_50_ values observed when comparing XTT to PrestoBlue were similar. On the HeLa cells the positive control showed IC_50_ values of 0.005 ± 2 × 10^−4^ *µ*g/mL and 0.006 ± 1 × 10^−4^ *µ*g/mL for XTT and PrestoBlue, respectively, whereas on the A431 cells the positive control showed IC_50_ values of  0.011 ± 2 × 10^−4^ *µ*g/mL for both growth indicators. The colour change was observed at approximately the same time and both growth indicators showed a distinguishable colour change.

PrestoBlue showed great potential as a growth indicator for various microorganisms as well as a cell viability. It has minor limitations such as light sensitivity and the varied time taken for colour development, which is dependent on the metabolic rates of various bacteria and cell cultures. Although these are potential drawbacks the results remain conclusive.

## 4. Conclusion

PrestoBlue and Alamar Blue give clear and easily determined visual MIC values for *S. mutans*, *P. intermedia*, *P. acnes*, and *M. tuberculosis.* However, *S. mutans, P. intermedia,* and *M. tuberculosis* developed a colour change more rapidly in PrestoBlue than in Alamar Blue. Furthermore Alamar Blue and PrestoBlue as growth indicator reagents have an advantage over other reagents, such as INT, in that the whole assay well changes colour instead of only the bacteria. Therefore PrestoBlue may be the better indicator for these microorganisms.

Comparing the reagents XTT and PrestoBlue as cell viability indicators it was observed that both growth indicators showed clear distinction between viable cells and nonviable cells and the IC_50_ values obtained from both reagents were similar.
